# Supplementary material for: Individual determinants of satisfaction with the work environment after relocation to activity-based workplaces: A prospective study
Source: PLoS One. 2023 Mar 31;18(3):e0281771. doi: 10.1371/journal.pone.0281771 (PMC10065282; doi:10.1371/journal.pone.0281771)
Supplement: Attachment — Submitted filename: review 100622.docx [file pone.0281771.s002.docx]

**Individual determinants of satisfaction with the work environment**

**after relocation to activity based workplaces: a prospective study**

This paper reports the results of associations between age, gender, education, occupational position, and office type before relocation - and changes in satisfaction with office design and the physical and psychosocial environment. Several of the investigated individual characteristics are found to predict changes in satisfaction from before to after relocation.

The following suggestions are offered to improve the paper:

1) Introduction, 1.1, page 4, first section: Please review if some of the past findings referred to might in fact overlap – does each publication need their own sentence? Also, the heading “1.1 satisfaction in activity based workplaces” should be considered changed as several of the studies referred to focuses on physical work environment, work engagement, health, psychosocial working conditions – aspects not equivalent to “satisfaction” per se. Please also consider offering a definition of “satisfaction”, alternatively an operational definition.
2) Introduction 1.2 second section page 5: It seems unclear why activity based work environments are discussed in relation to associations between “time on a particular job” and “satisfaction”. Is it to highlight that activity based work environments potentially might moderate impacts of time on a particular job on satisfaction?
3) 2.1 Please specify the statistical analysis for determining differences between responders and non-responders. And you might perhaps consider including the analysis in appendix.
4) The sentence “The questionnaire was constructed and administered using Webropol (..)” is written twice.
5) It is stated several places that certain aspects are associated with “less decreased satisfaction” but doesn’t the results actually show an *increase* in satisfaction (positive Beta values)? Also related to this, please check grammar and consider wording.
6) Please specify the purpose of the univariate analyses. It seems that you - regardless of significance/non-significance – adjust for all other variables in the multivariate models anyway.
7) Some consideration is given to why “education” suddenly becomes significant in the multivariate analyses compared the univariate analyses but it doesn’t seem to be sufficiently covered.
8) 3.3.3: It is stated below table 8 that - univariate analyses reveal that education is associated with the outcome - which does not seem to be the case given table 7.
9) How strongly do the three outcome measures correlate? Satisfaction with “the physical” environment and satisfaction with “the psychosocial” environment appears to be global general measures while satisfaction with workplace design taps into specific aspects of the physical and psychosocial environment. Thus, there seems to be (conceptually) a distinctive overlap between the outcome measures and it is not clear, in particular, what the analyses with the two global measures add.
