# Supplementary material for: Individual determinants of satisfaction with the work environment after relocation to activity-based workplaces: A prospective study
Source: PLoS One. 2023 Mar 31;18(3):e0281771. doi: 10.1371/journal.pone.0281771 (PMC10065282; doi:10.1371/journal.pone.0281771)
Supplement: Attachment — Submitted filename: Response to reviewers.docx [file pone.0281771.s003.docx]

**Response to reviewers**

**Individual determinants of satisfaction with the work environment after relocation to activity based workplaces: a prospective study**

Thanks for all comments, below you see how we have improved the manuscript in accordance with your comments.

Comments to each suggestion:

We have ensured that the manuscript meets the Plos Ones style requirement

The references has been changed to Vancocer style

Level 1 headings are bold type 18pt font, and level 2 heading bold type 16pt, level 3 14pt

Cited Figures written as Fig

Reviewer 1

**Comment:** A major problem of studying ABW is the definition of the concept. The present paper describes ABW on p 3: “In activity based workplaces employees share a non-assigned area designed for flexibility and different types of settings e.g. open meeting spaces, closed rooms, and silent spaces allowing different types of activities e.g. interaction with colleagues or silent concentration….” This can mean anything from landscapes with labelled zones to combinations of cell offices, meeting rooms, and remote work. Hence, important dimensions that may define how employees perceive and perform work are not defined: (a) number of employees in the room space, (b) area per employee, (c) fixed versus non-fixed workplace (shared seating), (d) number of workstations relative to number of employees (i.e., availability of preferred workstation, availability of workstation close to team members), (e) level of remote work.
The present paper does not define or describe the ABW studied, hence the external validity is limited. Did all employees have shared seating/clen desk? Did managers have the same work situation as all others in the ABW? Furthermore, the work tasks performed are not described. Time spent on solitary work, time spent in telephone conversations with clients or suppliers, and time spent in meetings define the needs of the employees. There is no description or data of these parameters and description of work tasks. There is no definition of or criteria for “private or shared office” or “open plan office” at baseline.

**Response:** This has been clarified in 2.1: Employees at the agency that did not relocate or had received customized workplaces were also excluded, meaning that all participants regardless of position or work tasks in the study work in activity based workplaces after relocation. The duties at the agency were mainly administrative, although the profession varied. The non-assigned area designed for flexibility and different types of activity was divided in labelled zones i.e. quiet zones and meeting rooms. Before the relocation it was ensured by a designated project group that the activity based workplace will be dimensioned to be enough for everyone. During the working day one are supposed to shift zone as tasks varies, meaning that the needs of the employee govern where one work. Before relocation a set of preparation activities i.e. education, information was conducted in order to ensure that everyone know office rules etc.

Further a defintion has been added: *Office type before relocation was divided in two categories, in both groups you had a dedicated seat. Private or shared office mean that one to four persons are seated in one cell office with a door that can be closed, while open plan office are larger and shared by many.*

We also add a referens to previous paper where this is more extensively described. Bergsten, E.L.; Wijk, K.; Hallman, D.M. Relocation to Activity-Based Workplaces (ABW)—Importance of the Implementation Process. Int. J. Environ. Res. Public Health 2021, 18, 11456. https://doi.org/10.3390/ ijerph182111456

**Comment:** The authors conclude that “Results from both univariate and multivariate models indicate that occupational position and office type before relocation predict the change in work environment satisfaction during relocation to activity based workplaces.” This is based on standard epidemiological analyses and there is no reason to doubt these findings. However, these analyses must be supplemented by specific information of the participants’ subgroups. For instance, the number of managers (or high-level of education) were working in individual offices at baseline. The paper should present the data showing the number of managers and employees, university degree and elementary etc training, age groups occupying the various office types at baseline, and their respective satisfaction reports. Data of baseline versus follow-up means (or medians) of all these subgroups would strengthen the quality and usability of information of the paper (even if some groups would be too small for testing statistical significance). **Response:** A new table (table 2) has been added with information on subgroups showing the number of managers and employees, university degree and elementary etc training, age groups occupying the various office types at baseline, and their respective satisfaction reports, showing mean, percent, number, SD . This table has been added in the result section 3. Previous table 1 has been changed and taken away from method section.

**Comment:** According to Figures 2 and 3, the mean levels of physical and psychosocial satisfaction ranged from 2.2 - to 2.7. These numbers are very low – are they correct?

**Respons:** In figure 2 and 3 we used three categories to illustrate the result. From reviewers feedback we relize that it was not clear, thanks. We have now changed the figures 2 and figure 3 so that the x and y contain the scale 1-5 instead of categories (mean value).

**Comment:** The Discussion contains references to studies of job satisfaction and of gender effects. The authors should either delete references that are not related to office concepts or do a comprehensive summary of the hundreds of studies in these fields.

**Response:** We have clarified in the discussion that the references on previous studies on gender and job satisfaction only are examples. We like to kep the references in order to discus our results in relation to prevous studies on gender and job satisfaction but do not se the neccesity of summarizing hundreds of studies.

**Comment:** The authors maintain that “Previous studies have shown that there is more communication in open offices, which might contribute to a better satisfaction among workers who need or like communication (Banbury & Berry, 2005; van der Voordt, 2004).” This is controversial and the recent study by Bernstein and Turban (Philosophical Transactions B, 2018) who showed with objective recording methods that face-to-face interactions decreased by ca 70%, should be included an discussed.

**Response:** We have added the reference in the discussion: Bernstein, Ethan, and Stephen Turban. ["The Impact of the 'Open' Workspace on Human Collaboration."](http://rstb.royalsocietypublishing.org/content/373/1753/20170239) Art. 239. Philosophical Transactions of the Royal Society B, Biological Sciences 373, no. 1753 (August 19, 2018).

**Comment:** The authors stated that “In future research, we encourage to further explore individual factors that can contribute to work satisfaction with respect to specific work tasks, and to explore the extent to which work related values contribute to work satisfaction in different groups of individuals.” A study by Nielsen and co-workers (Scand J Work Environ Health, 2021) reported effects of personality type and office type on disability retirement. The pronounced effects reported in that study illustrate the necessity to define criteria for defining a workplace as open-plan office versus ABW.

**Response:** This reference has been added and commented in the discussion

Reviewer 2

**Comment:** Introduction, 1.1, page 4, first section: Please review if some of the past findings referred to might in fact overlap. does each publication need their own sentence

**Response:** We have rewritten the introduction some and clarified that from previous studies we do not know if individual factors i.e. age, gender, education, working position and office type at baseline predict work satisfaction in activity based workplaces.

**Comment:** Also, the heading “1.1 satisfaction in activity based workplaces” should be considered changed as several of the studies referred to focuses on physical work environment, work engagement, health, psychosocial working conditions – aspects not equivalent to “satisfaction” per se.

**Response:** Title 1:1 has been changed

**Comment:** Please also consider offering a definition of “satisfaction”, alternatively an operational definition.

**Response:** A definition are added in 2.3: We used work environment satisfaction, focusing satisfaction with the physical and psychosocial work environment, as a measure of the overall perception of the work environment.

**Comment:** Introduction 1.2 second section page 5: It seems unclear why activity based work environments are discussed in relation to associations between “time on a particular job” and “satisfaction”. Is it to highlight that activity based work environments potentially might moderate impacts of time on a particular job on satisfaction?

**Response:** Thanks, this section in introduction 1.2 has been rewritten. What we want to describe is that “workers perception of work environment may change during ones working life”, inferring that age might contribute to perception of working in activity based work places.

**Comment:** 2.1 Please specify the statistical analysis for determining differences between responders and non-responders. And you might perhaps consider including the analysis in appendix.

**Response:** Thank you. We have rewritten and added a new table in the result section table 2 section. The table contain descriptive data; mean, number, percent and SD.

Since the response rate was high at baseline, the interesting thing to know would be if there are differences between respondents participating at both occasions, compared to those only answering at baseline, since we compare only those who had answered both times.. information in section 2.1 that the analysis was descriptive and that there were no major differences in terms of age, gender, education, position or office type among respondents that received questionnaires and did not answer after relocation, compared to those who did answer both

**Comment:** The sentence “The questionnaire was constructed and administered using Webropol (..)” is written twice.

**Response:** Thanks, this has been changed

**Comment:** It is stated several places that certain aspects are associated with “less decreased satisfaction” but doesn’t the results actually show an *increase* in satisfaction (positive Beta values)? Also related to this, please check grammar and consider wording.

**Response:** The results show decreases in satisfaction as described in 3.1, 3.2, 3.3. Nevertheless some persons in the group show less decreases than others, for instance, as described in 3.3.2, women show less decrease in satisfaction compared to men. This does not mean that satisfaction increase for woman but that the decreases were more pronounced for men. Proofreading has been conducted by a professional proofreader; you can see most of the changes in the manuscript.

**Comment:** Please specify the purpose of the univariate analyses. It seems that you - regardless of significance/non-significance – adjust for all other variables in the multivariate models anyway.

**Response:** Our aim was to determine the extent to which factors age, gender, education working position and office type at baseline predict satisfaction. Univariate analyses make it possible to specify the relationship between two variables, for instance age and satisfaction. We wished, however, to also see the composite relationship, using several variables as determinants. Those two analyses are complementary since univariate analyses do not allow for confounding factors to be taken into account

**Comment:** Some consideration is given to why “education” suddenly becomes significant in the multivariate analyses compared the univariate analyses but it doesn’t seem to be sufficiently covered.

3.3.3: It is stated below table 8 that - univariate analyses reveal that education is associated with the outcome - which does not seem to be the case given table 7.

**Response**: This is, as described above, an example of how univariate and multivariate analysis are complementary and give us additional information. The relationship between education and satisfaction are not significant, but when several variables are analyzed together, education seems to predict satisfaction. In the discussion, we have addressed this. We do not have information on why education predicts satisfaction.

**Comment:** How strongly do the three outcome measures correlate? Satisfaction with “the physical” environment and satisfaction with “the psychosocial” environment appears to be global general measures while satisfaction with workplace design taps into specific aspects of the physical and psychosocial environment. Thus, there seems to be (conceptually) a distinctive overlap between the outcome measures and it is not clear, in particular, what the analyses with the two global measures add.

**Response**: This is an interesting question. We believe that the measures contribute to valuable knowledge on self-reported perceptions about overall physical and psychosocial work environment while the six questions about workplace design ask more specifically about opportunity to reach colleagues, physical distance between colleagues, size of workplace, design and technical tools. All results show the same tendency, i.e. decreases in satisfaction after relocation, but we have not measured correlations between the variables in this paper. In another paper it could be interesting to measure correlation between different outcome measures, especially if the aim is to further validate measurements.

**Comment:** There is however no given definition of or criteria for «private or shared office» or «open plan office» at baseline.

**Respons:** A definition has been added in 2.3. In a ‘private or shared office’ one to four persons share one cell office with a door that can be closed, while an ‘open plan office’ is larger and shared by many. In the present study, workers had a personal working place in both office types.

**Comment:** A revised manuscript should should present the data showing the number of managers and employees, university degree and elementary training, age groups occupying the various office types at baseline,

**Response:**The number in each group working in private/shared office vs. open plan office before relocation has been added in a new table (table 2) in section 3.
