# Supplementary material for: Individual determinants of satisfaction with the work environment after relocation to activity-based workplaces: A prospective study
Source: PLoS One. 2023 Mar 31;18(3):e0281771. doi: 10.1371/journal.pone.0281771 (PMC10065282; doi:10.1371/journal.pone.0281771)
Supplement: Attachment — Submitted filename: Response to Reviewers - second revision.docx [file pone.0281771.s004.docx]

**Response to reviewers – second revision**

Thanks for all comments, below you see how we have improved the manuscript for the second time, in accordance with your comments.

Comments to each point raised:

**Reviewer #3**

***1. Introduction:*** *What process was followed to select the (five) specific independent individual worker characteristics/variables (i.e. baseline age, gender, education, working position and office type) to the exclusion of other potential individual characteristics such as personality type which may affect satisfaction with transition to ABW?*

**Response:** In section 2:1, we refer to previous studies (references 3, 19-32) indicating that job satisfaction in general may be related to age, gender, education, office type and seniority. On page 5, we argue that “Given that a growing body of literature emphasizes the influence of activity-based workplaces on work satisfaction in general, and the importance of individual factors in particular, we find it useful to further examine the extent to which different individual factors have an impact on work satisfaction in activity-based workplaces”.

We considered controlling for job title, but chose not to since tasks vary greatly within the professional categories; for example, administrators may carry out a wide variety of individual administrative tasks, including various interactive tasks. Personality would also have been interesting to study, but this would have required a different approach than a web survey. We have added this limitation to the methodological discussion in section 4:1

***2. Methods:*** *Was there any attempt in the survey to describe or define for respondents what they should understand by the terms Physical work environment and psychosocial work environment in answering the respective single item questions (“Regarding your work in general, how satisfied are your regarding the physical work environment?”, and "Regarding your work in general, how satisfied are your regarding the psychosocial environment"). These terms may mean different terms to different individuals, particular the psychosocial work environment. In contrast satisfaction with workplace design was limited specifically to 6 questions.*

**Response:** As mentioned in section 2:3, we asked for perceived overall satisfaction with physical and psychosocial work environment and did not define these concepts, which has been added as a limitation in the discussion. In section 2:3, we have also clarified that we asked for *perceived* overall work environment satisfaction.

***3. Results:*** *were mostly clear. However in Table 2 there is an error in percentage of elementary school education in the total sample (32.9.4). Additionally, Page 19 (line 1-2) incorrectly reports that".. both univariate and multivariate analyses reveal that education, position......associated with changes in perceived satisfaction with the psychosocial work environment during relocation to activity based workplaces". This finding is not the case for univariate analysis (see Table 3 and line 2-3 page 18).*

**Response:** Thank you, this mistake has been corrected in the manuscript in Table 2 and on page 19.

***4. Discussion:*** *Methodological limitation should acknowledge that some individual characteristics such as personality type were not included in the study as independent factors potentially influencing worker satisfaction.*

**Response:** Thank you for this comment. We agree that other factors, such as personality type, may be of importance, and this has been added to the discussion in section 4:1.

***5. Practical implications:*** *The statement "A workplace with, for example mainly managers may find it easier to gain acceptance for activity based workplaces, compared to a group of workers"; does not accord with the findings of the research or indeed the conclusion above on page 24 "employees showed less decrease in satisfaction than managers".*

**Response:** Thank you for this important comment. We have revised the manuscript by removing the sentence from the manuscript in order to make sure there will be no misunderstandings. What we intended to say is that employees were less satisfied compared to managers and that managers generally rated questions about satisfaction higher than employees did.

**Reviewer #4:**

*1.Although I recommended accepting the manuscript, however, the manuscript requires* ***proofread*** *by native English person. There are many simple structure errors and grammatical mistakes.*

**Response:** A professional proofreader has carefully corrected grammatical and structural errors, see track changes in manuscript.

*Have the authors made all data underlying the findings in their manuscript fully available?*

*Reviewer #3: No; Reviewer #4: Yes*

**Response:** The data contain sensitive information. Due to the ethical restrictions imposed by the Swedish Ethical Review Authority (at the time of the study: Regional Ethical Review Board in Uppsala), original data more detailed than the attached minimal dataset are available only upon request. Reasonable requests for data may be sent to the following email address, with due reference to the name of the project: [registrator@hig.se](mailto:registrator@hig.se).
