# Supplementary material for: Individual determinants of satisfaction with the work environment after relocation to activity-based workplaces: A prospective study
Source: PLoS One. 2023 Mar 31;18(3):e0281771. doi: 10.1371/journal.pone.0281771 (PMC10065282; doi:10.1371/journal.pone.0281771)
Supplement: Attachment — Submitted filename: Response to Reviewers.docx [file pone.0281771.s005.docx]

**Response to reviewers – final revision**

#The authors need careful attention to the presentation of the Tables 3-8 and make sure consistencies in the results of the statistical analysis and statements in the body of the manuscript. The authors may need to seek advice of experts of statistical analysis to make sure the interpretation of the results and academic presentation of the results of statistical analysis.

**Response:** Thanks, presentations of Tables 3-8 have been changed, se example of new tables below. An expert of statistical analysis has been consulted during the process.

|  | **B (95% CI)** | **Sig.** | **Adjusted R*^2^*** |
| --- | --- | --- | --- |
| **Age**:  *Years* | -0.014 (-0.028 to -0.001) | **0.038** | 0.010 |
| **Gender**  *ref female* | -0.296 (-0.553 to -0.039) | **0.024** | 0.012 |
| **Education**  *ref not university* | -0.051 (-0.333 to 0.231) | 0.722 | -0.002 |
| **Position**  *ref employee* | 0.588 (0.105 to 1.071) | **0.017** | 0.013 |
| **Office before relocation**  *ref cell office* | 0.571 (0.287 to 0.855) | **<0.001** | 0.035 |

#Journal Requirements: Please review your reference list to ensure that it is complete and correct. If you have cited papers that have been retracted, please include the rationale for doing so in the manuscript text, or remove these references and replace them with relevant current references. Any changes to the reference list should be mentioned in the rebuttal letter that accompanies your revised manuscript. If you need to cite a retracted article, indicate the article’s retracted status in the References list and also include a citation and full reference for the retraction notice.

**Response:** Some minor revisions have been made in the reference list, as you can see in the revised manuscript. One reference (Bourmans et al) has been removed since it was duplicated, therefore the reference numbers also changed throughout the manuscript.

#Reviewer #3: I am satisfied that the authors' response letter has addressed all the issues I raised in my last review, and are reflected in the second revised manuscript with the exception of one issue. I previously noted that on page 18 it is stated that no significant differences in changed satisfaction with the psychosocial work environment were found for education. However on page 19 the authors conclude in a statement that both univariate and multivariate analyses revealed that education...was associated with changes in perceived satisfaction with the psychosocial work environment during relocation to activity-based workplaces. This statement may be correct for all other variables but not education. Repetition of this error makes me question the proof reading of the manuscript by the authors given they indicated in their response that the error had been corrected. However, if this issue is addressed I am satisfied that the manuscript is ready for publication.

**Response:** Thanks for this comment, we have revised the manuscript.

#Reviewer #4: Thank you for addressing most of the comments. However, Table 3 to Table 8 still need amendments. We dont normally copy and paste the output from SPSS and put in the Table here. I suggest to report only important information, such as Mean (SD), effect size, significant only. Please see other journal in PLOS one on the Table reporting. Thank you.

**Response:** The tables have been revised and are no longer containing information on SE. We have also put confidence intervals for B in the same column as B to simplify the information. However, we believe that it is much more informative and more ‘modern’ to report the results of all variables in the table, even those that are not significant.
